# Significance of hanging total spine x-ray to estimate the indicative correction angle by brace wearing in idiopathic scoliosis patients

**DOI:** 10.1186/1748-7161-7-8

**Published:** 2012-03-27

**Authors:** Hiroshi Kuroki, Naoki Inomata, Hideaki Hamanaka, Etsuo Chosa, Naoya Tajima

**Affiliations:** 1 Department of Orthopaedic Surgery, University of Miyazaki Faculty of Medicine, Miyazaki, Japan; 2 Department of Orthopaedic Surgery, Nozaki Higashi Hospital, Miyazaki, Japan

**Keywords:** Idiopathic scoliosis, Hanging total spine x-ray, Spinal flexibility, Brace treatment, Osaka Medical College (OMC) brace

## Abstract

**Background:**

Although most idiopathic scoliosis patients subject to conservative treatment in daily clinical practice, there have been no ideal methods to evaluate the spinal flexibility for the patients who are scheduled the brace treatment. The purpose of this study was to investigate the value of hanging total spine x-ray to estimate the indicative correction angle by brace wearing in idiopathic scoliosis patients.

**Methods:**

One hundred seventy-six consecutive patients with idiopathic scoliosis who were newly prescribed the Osaka Medical College (OMC) brace were studied. The study included 14 boys and 162 girls with a mean age of 13 years and 1 month. The type of curves consisted of 62 thoracic, 23 thoracolumbar, 22 lumbar, 42 double major, 14 double thoracic, and 13 triple curve pattern. We compared the Cobb angles on initial brace wearing (BA) and in hanging position (HA). Of those, 108 patients who had main thoracic curves were selected and evaluated the corrective ability of OMC brace. These subjects were divided into three groups according to the relation between BA and HA (BA < HA group, BA = HA group, and BA > HA group), and then, maturity was compared among them.

**Results:**

The average Cobb angle in upright position (UA) of all cases was 31.0 ± 7.8°. The average BA and HA of all cases were 20.3 ± 9.5° and 21.1 ± 8.4°, respectively. The average chronological age was lowest in BA < HA group. And also, maturity in BA < HA group was the lowest among each of them. The rate of BA < HA cases were decreased as the Risser stage of the patients were progressed.

**Conclusions:**

The use of hanging total spine x-ray served as a useful tool to estimate the degree of correction possible curve within the OMC brace for main thoracic curve in idiopathic scoliosis. Maturity had some influence on the correlation between HA and BA. Namely, in immature patients, HA tended to be larger than BA. In contrast, in mature patients, HA had a tendency to be smaller than BA. With consideration for spinal flexibility based on maturity, in mature patients, larger BA than HA may be allowed. However, in immature patients, smaller BA than HA should be aimed.

## Introduction

Various types of stress radiographs to evaluate spinal flexibility in patients with scoliosis have been reported so far [1-12]. They help to determine the extent of structural change in the spine, the rigidity of a particular curve, and indicate the degree of correction safety possible with instrumentation and fusion. Of those, the first aim is to obtain information about the reducibility of deformity and deciding fusion levels for the patients who were scheduled surgical treatment. However, although most idiopathic scoliosis patients subject to conservative treatments in daily clinical practice [13], there have been no ideal methods to evaluate the spinal flexibility for the patients who are prescribed the brace. Brace treatment is the only conservative management to prove effectiveness [14-16] and the stiffness of the spine is an important factor that determines the positive outcome related to immediate in-brace correction [17]. Therefore, development of stress radiographs to evaluate spinal flexibility suitable for scoliosis patients who are indicated brace treatment is desirable. Undoubtedly, for the patients in juvenile or adolescent, opportunities and dosage of radiation should be reduced as much as possible. Whereas, some additional x-rays exposure to achieve appropriate management of curves must be acceptable under the condition of the maximum efforts to decrease dosage of radiation; shorter exposition time and coverage of gonads. We evaluate the flexibility of the spine in patients with idiopathic scoliosis by hanging total spine x-ray before Osaka Medical College (OMC) brace [18] treatment. This radiograph is easily taken in outpatient clinic without any expensive equipment, extra-time, and extra-workforce [19,20]. The purpose of current study was to investigate the usefulness of hanging total spine x-ray to assess the spinal flexibility for the estimation of the indicative correction angle by brace wearing in idiopathic scoliosis patients.

## Materials and methods

**Figure 1:**
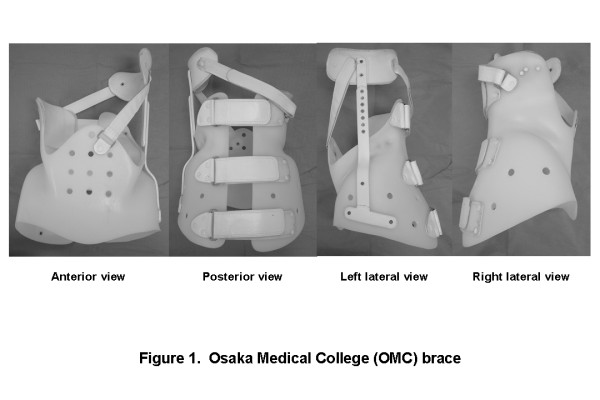
**Osaka Medical College (OMC) brace**. The OMC brace simply consists of a pelvic girdle, an upright bar, a high thoracic pad, and two straps. A pelvic girdle facilitates correction of a main thoracic curve. An upright bar with a high thoracic pad can correct a proximal thoracic curve utilizing righting reflex produced by patients' own response. Anterior and posterior straps prevent the body moving away from the brace during forward and backward bending motion.

From 1999 through 2007, 176 consecutive patients (258 curves) with idiopathic scoliosis who were newly prescribed the OMC brace and taken hanging total spine x-ray were studied. The OMC brace is an underarm brace developed by Onomura et al in the 1970s which features inconspicuous design, light weight, its reduced restriction on the chest wall movement, and ability to correct the high thoracic curve by righting reflex [18]. The concept of this brace is maintenance of whole body alignment and balance. For the achievement of these goals, step-by-step molding from pelvic girdle to high thoracic level with correcting lumbar and main thoracic curves is important to generate desirable corrective force based on the principle of three points lateral compression (Figure 1). The study included 14 boys and 162 girls ranging in age from 9 years 6 months to 17 years 9 months, with a mean age of 13 years and 1 month. The type of curves consisted of 62 thoracic, 23 thoracolumbar, 22 lumbar, 42 double major, 14 double thoracic, and 13 triple curve pattern.

**Figure 2:**
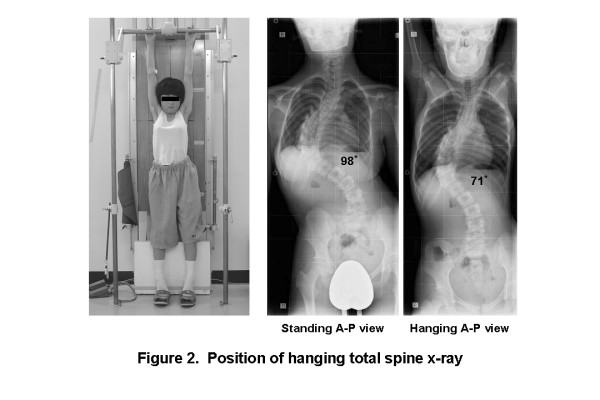
**Position of taking hanging total spine x-ray**. Hanging total spine x-ray was taken in position that the patient is hanging onto the bar, stretching the back, and touching the toes lightly to the floor not to sway the body. Patients were instructed making a great effort to stretch their back as much as possible.

Hanging total spine x-ray was taken in position that the patient is hanging onto the bar, stretching the back, and touching the toes lightly to the floor not to sway the body. Patients were instructed making a great effort to stretch their back as much as possible (Figure 2).

We compared among the Cobb angles in upright position (UA), on initial brace wearing (BA), and in hanging position (HA). Further, of those, 108 patients who had main thoracic curves were selected and evaluated the corrective ability of OMC brace. They included 13 boys and 95 girls ranging in age from 9 years and 6 months to17 years and 9 months, with a mean age of 13 years. These subjects were divided into three groups according to the relation between BA and HA (BA < HA group, BA = HA group, and BA > HA group), and then, maturity expressed by chronological age, duration after menarche, and Risser stage was compared among them. "BA < HA" was defined that BA is smaller than HA 5° or more, "BA = HA" was defined that difference between BA and HA is within 5°, and "BA > HA" was defined that BA is larger than HA 5° or more.

Statistical analysis was performed using two-tailed paired *t*-test and Pearson's correlation coefficient. P < 0.05 level was considered statistically significant.

Written informed consent was obtained from the patients for publication of this report and any accompanying images. And all procedures were in accordance with the Helsinki declaration.

## Results

**Figure 3:**
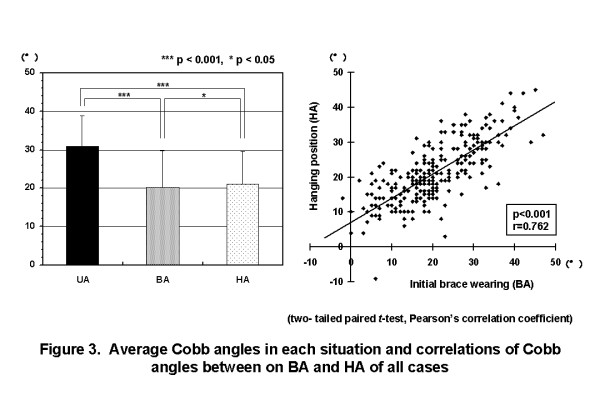
**Average Cobb angles in each situation and correlation between on initial brace wearing and in hanging position of all cases**. The average UA of all cases was 31.0 ± 7.8°. The average BA and HA of all cases were 20.3 ± 9.4° and 21.3 ± 8.4°, respectively. There were significant correlations between BA and HA. (UA: Cobb angle in upright position, BA: Cobb angle on initial brace wearing, HA: Cobb angle in hanging position).

**Figure 4:**
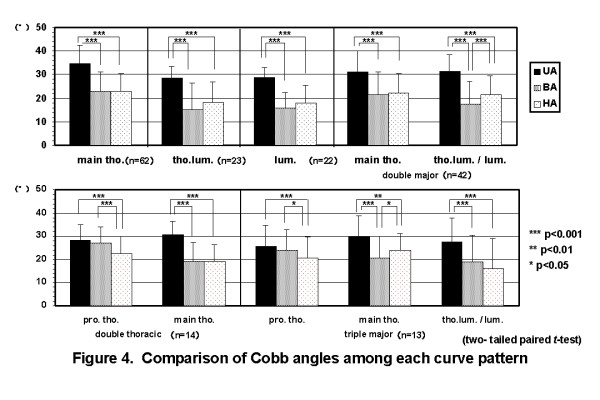
**Comparison of Cobb angles among each curve pattern**. In single curve patterns, BA was almost the same as HA. In multiple curve patterns, BA of major curves was almost the same as HA of them, except thoracolumbar and lumbar curves in double major curve pattern, proximal thoracic curve in double thoracic curve pattern, and proximal and main thoracic curves in triple major curve pattern. (BA: Cobb angle on initial brace wearing, HA: Cobb angle in hanging position).

The average UA of all cases was 31.0 ± 7.8°. The average BA and HA of all cases were 20.3 ± 9.5° and 21.1 ± 8.4°, respectively. There were significant correlations between BA and HA (Figure 3). In single curve patterns, BA was almost the same as HA. In multiple curve patterns, BA of major curves was almost the same as HA of them, except thoracolumbar and lumbar curves in double major curve pattern, proximal thoracic curve in double thoracic curve pattern, and proximal and main thoracic curves in triple major curve pattern (Figure 4).

**Figure 5:**
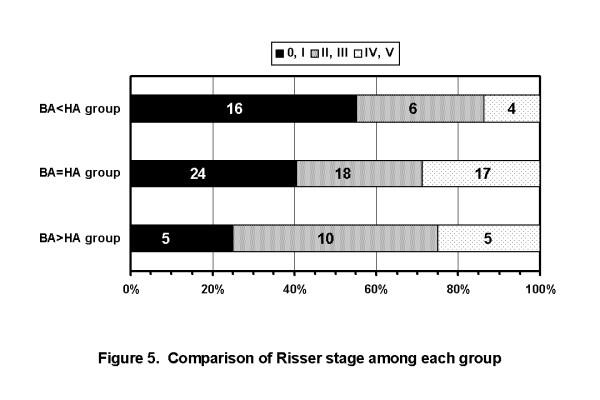
**Comparison of Risser stage among each group**. The rates of Risser 0 or I cases were 55.2% in BA < HA group, 40.7% in BA = HA group, and 25.0% in BA > HA group, respectively. Maturity in BA < HA group was the lowest among each of them. (BA: Cobb angle on initial brace wearing, HA: Cobb angle in hanging position).

**Figure 6:**
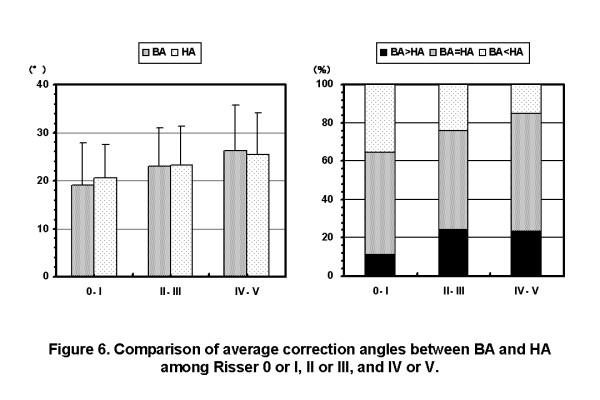
**Comparison of average Cobb angles between on initial brace wearing and in hanging position among Risser 0 or I, II or III, and IV or V**. In the patients of Risser 0 or I, the average BA was smaller than the average HA. While, in the patients of Risser IV or V, the average HA was smaller than the average BA. The rate of BA < HA cases were decreased as the Risser stage of the patients were progressed. (BA: Cobb angle on initial brace wearing, HA: Cobb angle in hanging position).

The average ages were 12 years and 9 months in BA < HA group, 13 years and 1 month in BA = HA group, and 13 years and 2 months in BA > HA group, respectively. The average durations after menarche were 4 months in BA < HA group, 8 months in BA = HA group, and 11 months in BA > HA group, respectively. The rates of Risser 0 or I cases were 55.2% in BA < HA group, 40.7% in BA = HA group, and 25.0% in BA > HA group, respectively. That is, the maturity of BA < HA group was lower than that of BA > HA group(Figure 5). In the patients of Risser 0 or I, the average BA was smaller than the average HA. While, in the patients of Risser IV or V, the average HA was smaller than the average BA. The rate of BA < HA cases were decreased as the Risser stage of the patients were progressed (Figure 6).

## Discussion

The assessment of spine flexibility has been traditionally performed using lateral-bending radiograph obtained with the patient in the supine or standing position [1]. In addition, push-prone radiograph in which the physician applies manual pressure on the apices of the curve [2,3], fulcrum-bending radiograph which is made with patient lying on his or her side over a large radiolucent plastic cylinder [4-6], supine traction radiograph which is obtained by applying traction force using a standard cervical traction halter with a second individual applying countertraction on both lower extremities [7,8], and suspension radiograph which was taken in lifting position by a axillary harness to create a spinal traction force resulting from the patient's own weight [9] are reported as stress radiographs to evaluate spinal flexibility in patients with idiopathic scoliosis. Further, some surgeons are employing general anesthesia to take supine traction radiograph for estimation of maximum curve flexibility just before surgery [10-12]. All of these stress radiographs are utilized as preoperative evaluation with the aim of predicting postoperative correction, planning fusion levels, and sparing patients anterior release surgery. At present, whereas, there is no report about stress radiographs for the patients with idiopathic scoliosis who are organized the conservative treatments. Because most idiopathic scoliosis patients subject to conservative treatments in daily clinical practice [13], it seems to be very important to develop an ideal method to evaluate the spinal flexibility for the patients who are indicated non-surgical treatments. In particular, it is so significant if the target angle corrected by brace will be able to set by some sort of stress radiographs for the idiopathic scoliosis patients since brace treatment is the most common and effective conservative treatment [14-16].

Immediate in-brace correction of scoliotic curve, 3D correction, and the absolute reduction of the Cobb angle are recognized to influence the treatments long-term effect [21]. Orthotists frequently consider that approximately 50% initial correction in the Cobb angle is necessary to expect a positive outcome [22,23]. Weiss et al [21] aim at an in-brace correction of more than 40% to make sure that for the patient the limitation of quality of life while brace wearing is worthwhile on the basis of the report from Landauer et al [24]. Castro et al suggested that brace treatment was not recommended in patients whose curves did not correct at least 20% in a TLSO [22]. The in-brace correction is dependent on curve pattern, age, sex, Cobb angle, and stiffness of the curve [25]. Accordingly, not all curves can be corrected to the same extent. An appropriate in-brace angle is thought to be different in individual cases according to these various conditions. So we require any stress radiographs that simply calculate an indicative correction angle by brace wearing on each patient.

Castro et al stated a side-bending radiograph to assess curve flexibility may be cost effective in preventing TLSO application to patients, with rigid curves, unlikely to benefit from its use [22]. However, all stress radiographs previously reported are not appropriate for evaluation of the patients who are performed conservative treatment since the mechanical force added to the spine of them is too much.

Then, we are routinely taking hanging total spine x-ray for the idiopathic scoliosis patients to assess if an appropriate correction by brace is achieved. HA were closely correlated with BA independent of curve patterns, except some curves in multiple curve patterns, and were useful for confirmation of adequate correction by brace.

In addition, we tried to find factors that affect the correction angle by OMC brace using main thoracic curve that is mostly common in idiopathic scoliosis. As the results of this investigation, maturity had some influence on the correlation between HA and BA. That is to say, in immature patients, HA tended to be larger than BA. In contrast, in mature patients, HA had a tendency to be smaller than BA.

Advantages of hanging total spine x-ray are as follows: it is easily taken in outpatient clinic without any expensive equipment, extra-time, and extra-workforce, the target angle corrected by OMC bracing can be estimated, and the condition of traction force stays constant because it depends just on the patient's own weight. Meanwhile, disadvantages of it are as follows: it is impossible to take for the patients who can not hang onto the bar, the force direction applied by this method is longitudinal which is not same as corrective force of OMC brace that is transverse, this method is not applied for some curves in multiple curve patterns, and some additional dosage of radiation can not be avoided.

Finally we have to supplementary say that the results of current study using the OMC brace do not always apply to those using other braces, especially braces of higher in-brace correction such as the Chêneau light brace [25,26]. Another research must be provided to confirm if hanging total spine x-ray will helpful or not for the treatment of idiopathic scoliosis using other braces.

## Conclusions

We conducted that in mature patients, larger BA than HA may be allowed, however, in immature patients, smaller BA than HA should be aimed. In spite of some limitations, we believe hanging total spine x-ray is useful for confirmation of adequate correction by the OMC brace in idiopathic scoliosis.

## Competing interests

The authors declare that they have no competing interests.

## Authors' contributions

HK conception and design, acquisition of data, analysis and interpretation data. NI acquisition of data. HH acquisition of data. EC analysis and interpretation data. NT analysis and interpretation data. All authors read and approved the final manuscript.
